# STIL-TA: A new model of traffic flow forecasting based on spatiotemporal interactive learning and temporal attention

**DOI:** 10.1371/journal.pone.0331095

**Published:** 2025-08-25

**Authors:** Linlong Chen, Linbiao Chen, Hongyan Wang, Jian Zhao

**Affiliations:** 1 School of Big Data & Information Engineering, Guiyang Institute of Humanities and Technology, Guiyang, China; 2 School of Computer & Communication, Lanzhou University of Technology, Lanzhou, China; 3 Big Data & Intelligent Engineering School & Chongqing College of International Business and Economics, Chongqing, China; School of Systems Science, Beijing Jiaotong University, CHINA

## Abstract

Accurate traffic flow forecasting plays a critical role in alleviating urban road congestion. Despite the success of existing models (e.g., graph-based or attention-based methods), three key limitations persist: (1) inflexible spatial dependency modeling, where static graph structures fail to adapt to dynamic traffic patterns; (2) decoupled spatiotemporal learning, where spatial and temporal correlations are processed separately, leading to information loss; and (3) limited long-term trend awareness, as traditional attention mechanisms overlook local contextual cues (e.g., rush-hour fluctuations). To address this, a new model of traffic flow forecasting based on Spatiotemporal Interactive Learning and Temporal Attention (STIL-TA) is proposed. This model effectively enhances the accuracy of traffic flow predictions by jointly modeling the spatiotemporal characteristics of road networks. Specifically, STIL-TA consists of two key components: (1) an interactive learning module built upon interactive dynamic graph convolution, which adopts a divide-and-conquer strategy to synchronize interactions and share the dynamically captured spatiotemporal features across different time periods, and (2) a temporal multi-head trend-aware self-attention mechanism, which utilizes local contextual information to transform the numerical sequence, enabling the capture of dynamic temporal dependencies in traffic flow and improving long-term prediction accuracy. Experimental results on four real-world traffic datasets demonstrate that the proposed STIL-TA model outperforms existing approaches, achieving significant improvements in forecasting accuracy.

## 1 Introduction

As urbanization accelerates, traffic problems have become increasingly severe, particularly in large cities. The continuous growth in traffic volume has led to the overload of road traffic systems and frequent congestion. To address this challenge, governments and research institutions worldwide have actively promoted the development and application of Intelligent Transportation Systems (ITS) [1], with accurate traffic flow prediction being regarded as a core component of ITS. Traffic flow prediction not only significantly enhances transportation efficiency but also effectively alleviates traffic congestion, reduces accident rates, minimizes energy consumption, and improves environmental quality.

Spatiotemporal traffic data, generated by sensors deployed at traffic network nodes, consists of sequential traffic data recorded at fixed time intervals. These data reflect the real-time operational state of the traffic network, making them a key data source for traffic flow prediction research. However, urban traffic conditions are influenced by various factors, and traffic flow exhibits high dynamic variation and uncertainty over time, posing significant challenges to accurate prediction. Therefore, the primary task in addressing traffic flow prediction is to identify effective methods that can capture the spatiotemporal data characteristics and handle its complex uncertainties.

Currently, the main approaches to traffic flow prediction can be categorized into three types: statistical methods, machine learning methods, and deep learning methods. Statistical methods capture the temporal characteristics of traffic flow through historical data analysis, with common models including the historical average model [2], the Autoregressive Moving Average (ARMA) model [3], and the Vector Autoregression (VAR) model [4]. For instance, Hamed et al. [5] proposed the ARIMA model for traffic flow prediction based on the analysis of historical data. However, statistical methods often struggle to handle nonlinear and high-dimensional complex data, leading to limitations in their predictive capabilities. To overcome these shortcomings, machine learning models have emerged, such as k-Nearest Neighbors (KNNs) [6] and Support Vector Regression (SVR) [7]. Mathew and Rawther [8] incorporated the correlation between traffic flows into the prediction by optimizing the k-NN classifier, while Wu et al. [9] applied SVR for travel time prediction, demonstrating its strong generalization ability. Nonetheless, machine learning models are still influenced by prior knowledge and parameter selection, making it difficult to guarantee consistent prediction performance.

In recent years, deep learning models, particularly deep belief networks (DBNs) and recurrent neural networks (RNNs), have shown significant potential in handling time-series data. Huang et al. [10] introduced a more accurate traffic flow prediction method by combining DBNs with regression models. RNNs [11,12] and their variants, such as Long Short-Term Memory (LSTM) [13] networks and Gated Recurrent Units (GRUs) [14], have become the mainstream approaches for time-series data due to their exceptional memory capabilities, achieving remarkable performance, especially in traffic flow forecasting. Despite the ability of statistical and machine learning methods to capture temporal characteristics of traffic flow effectively, they still exhibit considerable limitations when faced with high-dimensional traffic networks with spatiotemporal correlations [15].

Convolutional Neural Networks (CNNs) offer a novel solution to this problem. With their strong spatial feature extraction capabilities, CNNs help capture the spatial correlations within traffic networks. The integration of CNNs with RNNs has emerged as a promising approach to effectively model the spatiotemporal dependencies of traffic flow, becoming a hot topic in current research. For instance, Shi et al. [16] proposed a Convolutional LSTM (ConvLSTM) network that incorporates CNNs for extracting spatial features of road networks and combines them with LSTM to process temporal data, thus enabling spatiotemporal dependency modeling of traffic flow. Ke et al. [17] introduced the FCL-Net model, which combines ConvLSTM, standard LSTM, and convolutional layers, allowing it to address both spatiotemporal dependencies and external influencing factors. Zhang et al. [18], based on deep residual CNNs, proposed the ST-ResNet model for pedestrian flow prediction within urban areas, which demonstrated significant effectiveness.

Furthermore, many existing studies define various adjacency matrices to represent the deep structure of traffic flow in order to capture hidden dynamic spatial features. For instance, Wu et al. [19] use GCNs, 1D CNNs, and adaptive adjacency matrices to learn hidden spatial features, while Song et al. [20] combine multiple adjacency matrices with embedded structures for traffic flow prediction. Adaptive adjacency matrices explore hidden relationships between road network nodes to improve the model’s learning of the spatial heterogeneity of traffic flow. However, as training halts, adaptive adjacency matrices fail to learn the time-varying dynamic relationships between graph nodes. Despite the positive progress made by existing methods in capturing spatiotemporal features, there remains a deficiency in the interactive learning ability of dynamic spatiotemporal features. This limitation restricts the model’s capacity to perceive the periodicity, trend changes, and dynamic features of traffic flow, ultimately affecting prediction accuracy.

To address this challenge, we propose a novel traffic flow prediction model, STIL-TA, which significantly enhances prediction accuracy by fully exploiting the dynamic spatiotemporal features within traffic flow time series. First, we introduce a dynamic graph convolutional network (DGCN) that leverages prior knowledge to generate dynamic graphs, thereby capturing the latent spatial features of traffic flow. By embedding DGCN into an interactive learning framework, we construct the interactive dynamic graph convolutional network (IDGCN). This network analyzes the periodicity of traffic flow, segments the sequence into time intervals, and captures deep spatiotemporal dynamic features through interactive learning between subsequences. Additionally, we propose adaptive adjacency matrices and dynamic adjacency matrices to further uncover the time-varying dynamic relationships between nodes. To better handle the nonlinear temporal characteristics of traffic flow, we introduce a temporal multi-head trend-aware self-attention mechanism (TMHTAAtt) module, which effectively perceives local context and further integrates spatiotemporal features. By considering the periodicity and dynamic properties of traffic flow, STIL-TA overcomes the limitations of existing methods in modeling spatiotemporal dependencies, effectively capturing the dynamic spatiotemporal features of traffic flow and making it well-suited for traffic flow prediction tasks.

The main contributions are summarized as follows:

1. A novel spatiotemporal traffic flow prediction model, STIL-TA: This model embeds dynamic graph convolution into an interactive learning framework and introduces a new temporal multi-head trend-aware self-attention mechanism. The interactive learning structure captures spatiotemporal dependencies, while the self-attention mechanism further integrates spatiotemporal features, improving prediction accuracy.
2. A dynamic graph convolutional network is proposed to capture spatiotemporal features. The network generates a fusion of adaptive adjacency matrices and learnable adjacency matrices. The former captures the heterogeneity of traffic flow time series, while the latter learns the dynamic relationships between road network nodes.
3. An innovative temporal multi-head trend-aware self-attention mechanism is designed, enabling effective perception of local context and in-depth exploration of dynamic temporal relationships in traffic flow, further enhancing the model’s prediction accuracy.
4. Extensive comparative experiments on four traffic datasets demonstrate that the proposed model outperforms existing baseline methods in terms of prediction performance, achieving the best predictive results.

## 2 Related work

### 2.1 Traffic flow forecasting

Traffic flow forecasting has long been a critical research area within the field of transportation information systems. As one of the key issues in traffic management and optimization, it has attracted significant attention from the academic community. Over the past several decades, researchers have developed a variety of accurate traffic flow prediction models, utilizing both traditional mathematical models and data-driven approaches. Traditional mathematical models typically rely on statistical methods to analyze historical traffic data, assuming that future traffic flow shares certain similarities with past data, thereby enabling predictions.

With the continuous advancement of computational power and the rise of artificial intelligence technologies, traffic flow forecasting has once again become a focal point of research. Although traditional autoregressive models (e.g., ARIMA, VAR) and support vector regression (SVR) have shown promising results in certain applications, they often struggle to effectively handle non-stationary or complex time-series data, resulting in suboptimal forecasting performance in many real-world scenarios. In contrast to these traditional methods, deep neural networks and their variants, such as Long Short-Term Memory networks and Gated Recurrent Units, have demonstrated superior performance in capturing temporal dependencies within traffic flow data. These models are capable of extracting features from large volumes of sequential data and learning complex patterns. However, simple RNN models are still limited in their ability to leverage the spatial information inherent in traffic data. As a result, modeling spatial dependencies within spatiotemporal data has become a major challenge. To address this issue, researchers have explored the use of CNNs to capture spatial variations in Euclidean space. However, this approach is restricted to regular grid data. More recently, research has shifted towards the use of GCNs to model non-Euclidean relationships in road networks [21]. This method has shown significant potential in simulating the spatiotemporal dependencies of traffic flow, demonstrating its effectiveness in capturing the complex interactions within transportation systems [22,23].

Recently, Zhang et al. [24] propose the Event Flow Transformer Network (EF-former), a deep learning model for multi-step passenger flow prediction in Urban Rail Transit (URT) during large-scale events, using normal and extra outflow data to predict actual outflow and identify sudden passenger flow occurrences. Qiu et al. [25] propose a Spatial–Temporal Multi-Task Learning (STMTL) framework for predicting short-term passenger inflow and outflow in Urban Rail Transit (URT) systems during holidays. The framework includes a Multi-Graph Channel Attention Network (MGCA) that extracts and integrates both static and dynamic spatial dependencies from inter-station interaction graphs. Zhang et al. [26] propose a Multi-Frequency Spatial-Temporal Graph Neural Network (MFST-GNN) for accurately predicting metro Origin-Destination (OD) demand during public health emergencies. The model leverages multiple OD demand patterns, including real-time, daily, and weekly, to capture periodic spatial-temporal features. It includes a multi-frequency temporal feature extraction module for periodic temporal features and an adaptive spatial feature extraction module for complex hidden spatial features. Zhang et al. [27] propose a unified framework named Physics-Guided Adaptive Graph Spatial-Temporal Attention Network (PAG-STAN) for predicting metro Origin-Destination (OD) demand under pandemic conditions. Specifically, PAG-STAN includes a real-time OD estimation module to estimate complete real-time OD demand matrices and a novel dynamic OD demand matrix compression module to generate dense real-time OD demand matrices.

In recent years, the application of Large Language Models (LLM) [28] in the field of spatio-temporal data analysis has made significant progress, especially in the task of traffic flow prediction, where researchers have significantly improved the prediction accuracy and interpretability of the models through techniques such as fine-tuning, dynamic modeling, and multimodal alignment. For example, Zhao et al. [28] propose a novel method named Large Language Model Enhanced Traffic Flow Predictor (LEAF) to improve traffic flow forecasting by integrating LLM. LEAF consists of two branches: one using graph structures and the other using hypergraph structures to capture different spatio-temporal relationships. These branches are pre-trained separately and generate different predictions during testing. A large language model is then employed to select the most likely prediction result. Additionally, a ranking loss is applied as the learning objective to enhance the prediction capabilities of both branches. Guo et al. [29] propose a traffic flow prediction model named xTP-LLM, which leverages LLM to generate explainable traffic predictions. The model converts multi-modal traffic data into natural language descriptions to capture complex time-series patterns and external factors. The LLM framework is fine-tuned with language-based instructions to align with spatial-temporal traffic flow data.

### 2.2 Graph Convolution Networks

The Graph Convolutional Networks (GCNs) is an innovative technique that extends traditional convolution methods to graph-structured data. GCN approaches are generally classified into two main types: one that generalizes spatial neighborhood aggregation through convolutional filters, with the key challenge being the selection of neighboring nodes. A seminal work in this area is the graph convolution method based on attention mechanisms, proposed by Veličković et al. [30]. This method enhances the flexibility and expressive power of graph convolutions by assigning different weights to neighboring nodes. Another approach extends the convolution operation to the spectral domain via Fourier transforms, thereby enabling the processing of graph-structured data. For instance, Kipf and Welling [31] further simplified this approach, proposing the Graph Convolutional Network (GCNs), which optimizes the processing efficiency of graph data. With the continuous evolution of GCN technology, many improved models have emerged. For example, Li et al. [22] proposed the DCRNN model based on ChebNet, which effectively learns the spatial diffusion process of traffic flow data. Wu et al. [32] proposed an adaptive adjacency matrix that learns the graph structure and captures temporal dependencies through a data-driven approach. Additionally, Guo et al. [33] introduced a spatiotemporal attention mechanism that significantly enhances the model’s ability to learn dynamic spatiotemporal features.

The initial development of graph convolutions was marked by the work of Bruna et al. [34], who proposed a general graph convolution architecture based on the graph Laplacian operator. This laid the foundation for the field. Later, Defferrard et al. [35] introduced a Chebyshev polynomial approximation in graph theory, successfully circumventing the high computational cost associated with calculating the Laplacian eigenvectors, and achieved significant results. Building upon this, Yu et al. [36] proposed a gated graph convolutional network (GCN) specifically designed for traffic flow prediction. However, this model struggled to fully capture the dynamic spatiotemporal correlations present in traffic data.

In contrast, spatial-domain graph convolution [20] employs inductive learning to directly extract spatial features of nodes in the graph, as well as the spatial features of dynamically changing graphs. For example, Hechtlinger et al. [37] proposed the Graph CNNs model, where the convolution operation is defined as constructing neighborhoods using a random walk method and selecting a fixed number of neighboring nodes based on their expected size to build the neighborhood. Hamilton et al. [38] performed a certain number of samples on adjacent nodes and used an aggregation function to gather information from neighboring nodes to predict the node’s value. Although these methods have made certain improvements in capturing the dynamic spatiotemporal features of traffic flow, they still exhibit poor interactive learning capabilities in the spatiotemporal modules that extract dynamic spatiotemporal features. This limitation affects the ability of traffic flow prediction models to perceive the periodicity and trend variations of time series and results in insufficient capture of the dynamic spatiotemporal features of traffic flow. Chen et al. [39] propose a Time-Aware Structural Semantic Coupled Graph Network (TASSGN) that learns both structural and semantic features of graphs through a new graph learning module, captures temporal features with a self-sampling method and a time-aware graph encoder, and generates sparse graphs to capture unique node features. Jiang et al. [40] propose a recurrent network utilizing a memory network and incorporate this concept into the Meta-Graph Convolutional Recurrent Network (MegaCRN) by integrating a Meta-Graph Learner, enhanced by a Meta-Node Bank, into the GCRN encoder-decoder framework. Lai et al. [41] propose a Long-term Explicit–Implicit Spatio-Temporal Network (LEISN) for traffic flow prediction. This network features a Long-term Dependency Module to store hidden states from multiple previous time steps and utilizes two graph convolution-based branches to extract explicit and implicit spatial features. The fusion of all these features enables the prediction of the next state.

### 2.3 Attention mechanism

The attention mechanism has gained widespread application in various fields such as natural language processing, traffic flow prediction, and speech recognition due to its flexibility and efficacy in modeling complex dependencies. The core concept of attention is to identify and focus on the most relevant portions of the vast amounts of data, thereby enhancing the model’s expressive power and predictive performance. For instance, Liang et al. [42] introduced a multi-layer attention mechanism designed to model the dynamic spatiotemporal correlations between geographical sensors, effectively addressing the challenges of spatiotemporal data prediction. However, this approach often requires the training of a separate model for each time series, which can be computationally expensive. Guo et al. [33] proposed a graph convolutional network with an attention mechanism, which achieved promising results in traffic flow prediction.

In contrast to previous methods, this paper considers both the graph structure of traffic networks and the dynamic spatiotemporal characteristics of traffic data. We propose a novel temporal multi-head trend-aware self-attention mechanism, which not only captures the dynamic dependencies within the traffic network but also generates more effective feature representations. This, in turn, provides enhanced modeling capabilities for more accurate traffic flow prediction.

## 3 Methodology

### 3.1 Problem definition

The traffic network can be represented as a weighted directed graph G=(V,E,A) , where |V|=N denotes the set of nodes (representing the sensors in the traffic network), and E is the set of edges connecting all nodes in V (indicating the strength of connections between nodes). The graph G is represented by the spatial adjacency matrix $A\in {\mathbb{R}}^{N\times N}$, where $A_{ij}$ denotes the proximity (measured by the similarity of node features) or distance (measured by the Euclidean distance between road sensors) between nodes $v_{i}$ and $v_{j}$. Specifically, if $v_{i},v_{j}\in V$ and $\left(v_{i},v_{j}\right)\in E$, then $A_{ij}=1$; otherwise, $A_{ij}=0$. The traffic state at time step t is represented as a graph signal $X_{G}^{\left(t\right)}\in {\mathbb{R}}^{N\times C}$ on the traffic network G, where each element corresponds to C traffic features (e.g., speed, flow, etc.) observed by the respective sensors. Traffic flow prediction is a typical time series forecasting task. Formally, given the velocity observations at N nodes over the past T time steps, this can be represented as $\text{P}=\left[X_{G}^{\left(t-T+1\right)},X_{G}^{\left(t-T+2\right)},\cdots ,X_{G}^{\left(t\right)}\right]\in {\mathbb{R}}^{N\times C\times T}$. The objective is to predict the traffic flow velocities at all nodes over the subsequent T′ time steps, denoted as $Q=\left[X_{G}^{\left(t+1\right)},X_{G}^{\left(t+2\right)},\cdots ,X_{G}^{\left(t+T\prime \right)}\right]\in {\mathbb{R}}^{N\times C\times T\prime }$. Based on this notation, the traffic prediction problem is generally defined as:


Q=ℓ(P;G) (1)

where ℓ is the function to be learned.

### 3.2 Framework of STIL-TA

**Fig 1:**
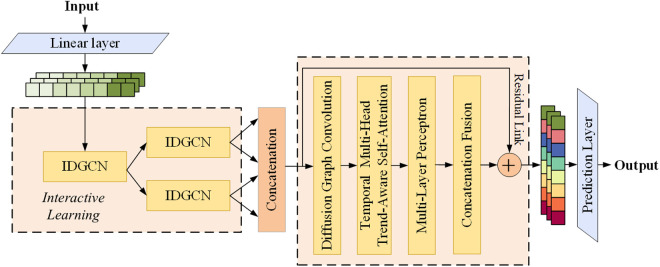
Overall Framework of STIL-TA.

This paper proposes a novel approach, STIL-TA, designed to simultaneously capture the dynamic spatiotemporal correlations of traffic flow. The overall framework of the model is illustrated in Fig 1, which primarily consists of an interactive dynamic graph convolutional network (IDGCN), a concatenation fusion module, and a temporal multi-head trend-aware self-attention (TMHTAAtt) mechanism. First, the raw data is fed into the Linear layer, which generates a high-dimensional spatial representation of the data to capture deeper spatiotemporal dependencies. Next, based on dynamic graph convolutional networks (DGCN), the IDGCN further processes the features extracted by the start convolution layer through an interactive learning strategy. Specifically, during the partitioning phase, the input data is split into two equal-length subsequences (each having half the length of the original sequence) via an interleaved sampling recursive method. These two subsequences are then subjected to interactive learning in the IDGCN, where the model shares the features learned by each subsequence. By embedding the DGCN into the interactive learning structure, the model not only captures temporal dependencies but also effectively learns the dynamic spatial characteristics of traffic flow. Once the spatiotemporal features are extracted by the IDGCN, the model outputs the two subsequences. Subsequently, these subsequences are reordered based on their temporal indices and are input into the diffusion graph convolution layer through the concatenation fusion module, further extracting the global dynamic spatiotemporal features of the traffic flow. Finally, the captured dynamic spatiotemporal features are passed to the TMHTAAtt mechanism and a multilayer perceptron layer for the final traffic flow prediction.

### 3.3 Interactive Learning (IL)

**Fig 2:**
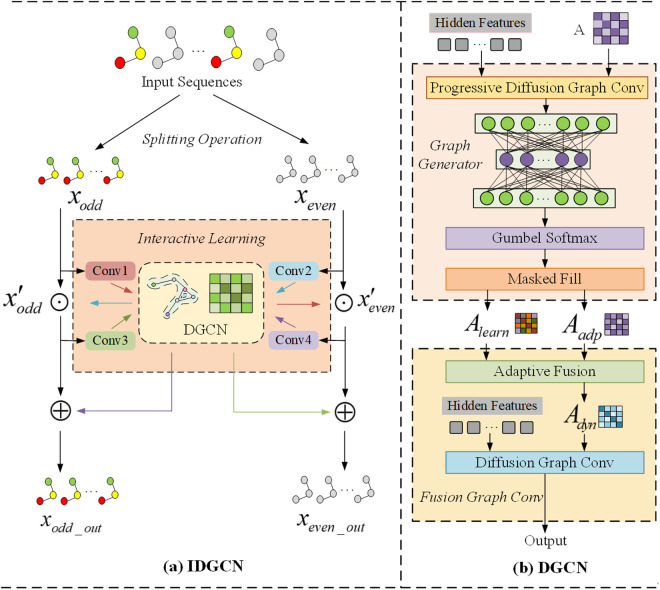
Overall Framework of the Interactive Learning Module: (a) Structure of the IDGCN; (b) Structure of the DGCN.

The interactive learning framework consists of three identical interactive dynamic graph convolutional network (IDGCN) modules, with the core component being the IDGCN structure, as illustrated in Fig 2a. The mechanism of the interactive learning framework is to capture the respective dynamic spatio-temporal features through the interactive learning of two subsequences. Each subsequence is first preprocessed by a convolution operation, and then these two subsequences share parameter weights within the IDGCN, which allows them to efficiently capture the dynamic spatio-temporal dependencies between them. This design allows the model to flexibly adjust its learning focus over different time periods, better adapting to the nonlinear and nonsmooth characteristics of time series.

The mathematical formulation of the interleaved sampling recursive method is as follows, given an input sequence $X\in {\mathbb{R}}^{T\times N\times F}$ (T time step, N nodes, F features), the sampling process is:


$$\left\{ \begin{array}{c} X_{even}=X\left[:,::2,:\right]\left(Even\;indexed\;time\;step\right)\\ X_{odd}=X\left[:,1::2,:\right]\left(Odd\;indexed\;time\;step\right) \end{array}\right.$$ (2)

where $X_{even}$, $X_{odd}\in R^{T/2\times N\times F}$, this nonoverlapping division preserves the integrity of the original sequence while enabling the two subsequences to capture complementary spatiotemporal patterns through interactive learning.

Let the input to IDGCN be denoted as $X\in {\mathbb{R}}^{T\times N\times F}$ is interlaced sampled to obtain two subsequences (odd and even sequences), $X_{odd}\in {\mathbb{R}}^{T/2\;\times N\times F}$ and $X_{even}\in {\mathbb{R}}^{T/2\;\times N\times F}$. The first stage of interactive learning produces outputs $\begin{array}{c} {X^{\prime }}_{odd}\in {\mathbb{R}}^{T/2\;\times N\times F} \end{array}$ and ${X\prime }_{even}\in {\mathbb{R}}^{T/2\;\times N\times F}$. Through further interaction, the final output sequences are ${X\prime }_{odd\_out}\in {\mathbb{R}}^{T/2\;\times N\times F}$ and $X_{even\_out}\in {\mathbb{R}}^{T/2\;\times N\times F}$. The specific operations in IDGCN are as follows:


$$X_{even},X_{odd}=Split\left(X\right)$$ (3)

$$X\prime_{odd}=tanh\left(DGCN\left(Conv1\left(X_{even}\right)\right)\right)⊙X_{odd}$$ (4)

$$X\prime_{even}=tanh\left(DGCN\left(Conv2\left(X_{odd}\right)\right)\right)⊙X_{even}$$ (5)

$$X_{odd\_out}=X\prime_{odd}+tanh\left(DGCN\left(Conv3\left({X\prime }_{even}\right)\right)\right)$$ (6)

$$X_{even\_out}=X\prime_{even}+tanh\left(DGCN\left(Conv4\left({X\prime }_{odd}\right)\right)\right)$$ (7)

where Conv1, Conv2, Conv3, and Conv4 represent 1D convolution operations, tanh denotes the activation function and the Hadamard product, and DGCN refers to the dynamic graph convolution in IDGCN.

The DGCN parameter $\theta_{DGCN}$ shared by the parity subsequence produces coupled gradient updates:


$$\nabla_{\theta }\text{L}=\frac{\partial \text{L}}{\partial {X\prime }_{odd}}\frac{\partial \tanh(DGCN(X_{even}))}{\partial \theta }+\frac{\partial \text{L}}{\partial {X\prime }_{even}}\frac{\partial \tanh(DGCN(X_{odd}))}{\partial \theta }$$ (8)

When the ratio is out of range, the anomalous gradient is automatically attenuated by the gating factor tanh(·).

Interleaved sampling ensures that both subsequences come from the same data stream shape:


$$X_{odd}\cup X_{even}=X,X_{odd}\cap X_{even}=\emptyset$$ (9)

The DGCN must accommodate both odd/even subsequence distributions, and its parameter $\theta_{DGCN}$ converges to the “intersection space” of the two substreams.

Compared with many existing models, the interactive learning framework offers the following improvements and unique advantages: first, it captures spatio-temporal dependencies more effectively by processing two subsequences in parallel. Second, the model is able to adjust the learning focus according to different time periods, better adapting to the dynamic changes of time series. Third, by sharing parameter weights, the model is able to integrate information from different time steps more effectively and improve learning efficiency. Finally, the model is able to better identify and utilize the periodic features in the time series while adapting to the continuous time pattern through the interactive learning mechanism.

### 3.4 Dynamic Graph Convolution Network (DGCN)

The dynamic graph convolutional network (DGCN) primarily consists of the diffusion graph convolution network and the graph generation module, as illustrated in Fig 2b. By leveraging the diffusion graph convolution and the graph generation module, DGCN effectively captures deeper dynamic spatial features, thereby enhancing the ability of STIL-TA to capture spatial heterogeneity. DGCN feeds the hidden feature matrix $H\in {\mathbb{R}}^{T\times N\times F}$ and a predefined initial adjacency matrix $A\in {\mathbb{R}}^{N\times N}$ into the diffusion graph convolution network, followed by feeding the output to the graph generator to produce a discrete matrix $A\prime \in {\mathbb{R}}^{N\times N}$ containing spatiotemporal information. The representation is as follows:


$$\begin{array}{c} A^{\prime }=Softmax\left(MLP\left(GCN\left(H,A\right)\right)\right) \end{array}$$ (10)

where GCN represents the diffusion convolution and graph generator operations, and MLP denotes a multilayer perceptron.

To ensure differentiability during training, the STIL-TA model employs the gumbel reparameterization:


Alearn=GumbelSoftmax(A′)=Softmax((log(A′)−log(−log(g)))/(log(A′)−log(−log(g)))τ\nulldelimiterspaceτ) (11)

where g~Gumbel(0,1) denotes a random variable, τ is the softmax temperature set to 0.5, and $A_{learn}$ is the adjacency matrix generated by the graph generator to model the dynamic dependencies between nodes. The temperature parameter τ is subjected to an exponential decay strategy (initial value of 0.5, 15% decay every 10 epochs) in order to progressively strengthen the discretization. The random variable g is generated by inverse transformation sampling to ensure that the gradient is computable during backpropagation.

Furthermore, an adaptive adjacency matrix $A_{apt}\in {\mathbb{R}}^{N\times N}$, which does not require any prior knowledge, is constructed as follows:


$$A_{apt}=Softmax\left(Relu\left(E_{1}E_{2}^{T}\right)\right)$$ (12)

where $E_{1}$ and $E_{2}^{T}$ represent learnable parameters, and the initial value of $A_{apt}$ is based on the adjacency matrix $A\in {\mathbb{R}}^{N\times N}$ derived from the original graph data.

DGCN uses an adaptive fusion module to merge $A_{learn}$ and $A_{apt}$, producing a dynamic adjacency matrix $A_{dyn}\in {\mathbb{R}}^{N\times N}$, which is then fed into the diffusion graph convolution network to extract hidden dynamic spatiotemporal correlations in traffic roads. The operation of this fusion module is as follows:


$$A_{dyn}=\alpha A_{apt}+\left(1-\alpha \right)A_{learn}$$ (13)

where α is a learnable adaptive parameter factor.

By gradient descent optimization with L2 regularization (λ=0.01), the update process can be expressed as:


$$\alpha_{t+1}=\alpha_{t}-\eta \left(\frac{\partial L}{\partial \alpha }+2\lambda \alpha_{t}\right)$$ (14)

where L is the loss function and η is the learning rate.

In the graph generator network, the fusion graph convolution, and the concatenation fusion module, diffusion graph convolutions are applied, and the input to the diffusion graph convolution is uniformly defined as $X_{in}\in {\mathbb{R}}^{C\times N\times T}$.

In the graph generator network, the diffusion graph convolution is defined as:


$$GCN\left(X_{in},A_{apt}\right)=\sum_{k=0}^{K}A_{apt}^{k}X_{in}W$$ (15)

where k represents the diffusion step, K is the maximum diffusion step, and W denotes the parameter matrix.

In the fusion graph convolution module, $A_{dyn}$ is the input adjacency matrix for the fusion graph convolution, where the diffusion graph convolution is represented as:


$$GCN\left(X_{in},A_{dyn}\right)=\sum_{k=0}^{K}A_{dyn}^{k}X_{in}W$$ (16)

In the concatenation module, the dynamic spatiotemporal features extracted by the IL module are recombined in temporal order and fed into the diffusion graph convolution layer to capture and correct features across the entire time series. Additionally, the forward and backward transition matrices Pf=A/Arowsum(A)\nulldelimiterspacerowsum(A) and Pb=AT/ATrowsum(AT)\nulldelimiterspacerowsum(AT) of the initial adjacency matrix A are used. The diffusion graph convolution in the concatenation fusion module is represented as:


$$GCN\left(X_{in},A,A_{dyn}\right)=\sum_{k=0}^{K}\left(A_{f}^{k}X_{in}W_{1}+A_{b}^{k}X_{in}W_{2}+A_{dyn}^{k}X_{in}W_{3}\right)$$ (17)

### 3.5 Temporal multi-head trend-aware self-attention

To capture the complexity and trend of traffic flow, we propose a temporal multi-head trend-aware self-attention (TMHTAAtt) mechanism that incorporates local contextual information. Traditional self-attention is a specific implementation of the attention mechanism, where queries, keys, and values are derived from the same symbol representation sequence. The multi-head self-attention mechanism is the most widely used variant in practice, as it enables simultaneous attention to information from multiple representation subspaces. The fundamental operations in multi-head self-attention are as follows:


$$Attention\left(Q,K,V\right)=Softmax\left(\frac{QK^{T}}{\sqrt{d_{model}}}\right)V$$ (18)

where Q represents the query, K represents the key, and V represents the value.

In multi-head self-attention, the query, keys, and values are initially projected into separate subspaces. The attention functions are then executed in parallel. The resulting outputs are concatenated and further projected to obtain the final output, represented as follows:


$$SelfAttention\left(Q,K,V\right)=⊕\left(head_{1},...,head_{h}\right)W^{O}$$ (19)

$$head_{j}=Attention\left(QW_{j}^{Q},KW_{j}^{K},VW_{j}^{V}\right)$$ (20)

where h represents the number of attention heads, $W_{j}^{Q}$, $W_{j}^{K}$, and $W_{j}^{V}$ are the projection matrices used on Q, K, and V, respectively, and $W^{O}$ represents the final output projection matrix. Multi-head self-attention offers a flexible approach to capture complex correlation dynamics in traffic data, leading to accurate long-term forecasting.

However, the multi-head self-attention mechanism was initially designed to handle discrete tokens and does not account for the inherent local trend information in continuous data. Therefore, directly applying it to traffic signal sequence transformation may lead to mismatching issues. TMHTAAtt addresses the local trend unawareness problem of traditional multi-head self-attention in numerical data prediction. This mechanism is a variant of convolutional self-attention. By employing convolutional operations, which compute representations based on local context as input, the model is able to capture the underlying local variation trends present in traffic data. Formally, the TMHTAAtt mechanism is defined as follows:


$$Trendhead_{j}=Attention\left(Q*\Phi_{j}^{Q},K*\Phi_{j}^{K},VW_{j}^{V}\right)$$ (21)

where * represents the convolution operation, while $\Phi_{j}^{Q}$ and $\Phi_{j}^{K}$ represent the parameters of the convolution kernel.

### 3.6 Other components

Huber loss [43] is a widely used loss function that combines the mean squared error (MSE) and the linear term of absolute error. It behaves like squared loss when the predicted values are close to the true values and like absolute loss when the predicted values are far from the true values. This characteristic allows huber loss to effectively mitigate the impact of outliers during model training while maintaining stable performance. Consequently, Huber loss is employed as the loss function in the optimization process of this study.


$$\begin{array}{c} \xi =\frac{1}{N\times Q}\sum_{i=1}^{Q}\sum_{j=1}^{N}R\left({\hat{x}}_{i,j},x_{i,j}\right)\\ R\left(\hat{x},x\right)=\left\{ \begin{array}{c} \frac{1}{2}{\left(\hat{x}-x\right)}^{2},\left|\hat{x}-x\right|\leq \delta \\ \delta \left|\hat{x}-x\right|-\frac{1}{2}\delta^{2},\left|\hat{x}-x\right|>\delta \end{array}\right. \end{array}$$ (22)

where δ is the hyperparameter that balances the Squared Error, $\hat{x}$ and x are the predicted and true values, respectively.

## 4 Experiment

### 4.1 Datasets

The predictive performance of the STIL-TA model was evaluated on two publicly available traffic datasets: METR-LA, PEMS-BAY, PEMS04 and PEMS08. METR-LA consists of traffic speed statistics recorded by 207 sensors located across highways in Los Angeles County over a four-month period. PEMS-BAY, on the other hand, comprises traffic speed data collected by 325 sensors deployed along roadways in the San Francisco Bay Area over a six-month span. Both datasets include information on the sensor locations, the dates of data collection, and the types of data recorded. PEMS04 and PEMS08 are real-world traffic datasets collected in real-time every 30 seconds by the California Department of Transportation’s Performance Measurement System (PeMS). A detailed description of the experimental datasets is provided in Table 1.

**Table 1:** Details of the experimental dataset.

Data	METR-LA	PEMS-BAY	PEMS04	PEMS08
Type	sequentially	sequentially	sequentially	sequentially
Attribute	speed	speed	flow	flow
Location	Los Angeles	the Bay Area	San Francisco Bay Area	Los Angeles
Edges	1515	2369	340	259
Time Steps	34272	52116	16992	17856
Nodes	207	325	307	170
Time Range	03/2012-06/2012	01/2017-05/2017	01/2018-02/2018	07/2016-08/2016

### 4.2 Parameter setting

In the experiment, historical traffic data from the past hour (T=12) were used to predict the traffic flow for the next 60 minutes (T′=12). The entire dataset was split into training, validation, and test sets in a 6:2:2 ratio, maintaining chronological order. The traffic flow was predicted for 15, 30, and 60-minute intervals. The batch size was set to 64, the learning rate was set to 0.001, dropout was set to 0.3, epoch was set to 500, weight decay was set to 0.0001, and the Python version was 3.6.2. All models were trained for 200 epochs using the Adam optimizer. All experiments were performed on a 22 vCPU AMD EPYC 7T83 64-Core Processor with a RTX 4090 GPU Card. Model performance was evaluated using three metrics: Mean absolute error (MAE), root mean square error (RMSE), and mean absolute percentage error (MAPE), as follows:


$$MAE=\frac{1}{F}\sum_{i=1}^{F}\left|{\hat{\partial }}_{i}-\partial_{i}\right|$$ (23)

$$MAPE=\frac{1}{F}\sum_{i=1}^{F}\left|\frac{{\hat{\partial }}_{i}-\partial_{i}}{{\hat{\partial }}_{i}}\right|\times 100\%$$ (24)

$$RMSE=\sqrt{\frac{1}{F}\sum_{i=1}^{F}{\left({\hat{\partial }}_{i}-\partial_{i}\right)}^{2}}$$ (25)

where F represents the number of samples, and ${\hat{\partial }}_{i}$ and $\partial_{i}$ denote the predicted and ground truth values for the i -th sample, respectively. The smaller the values of MAE, RMSE, and MAPE, the better the performance of the STIL-TA model in traffic flow prediction.

### 4.3 Baselines

To evaluate the performance of STIL-TA, we compare it with the following models:

● HA [2]: The Historical Average model, which predicts traffic flow based on the historical average traffic data.
● VAR [4]: The Vector Auto-Regression model, a statistical model that captures the linear relationships among multiple time series.
● SVR [7]: Support Vector Regression (SVR) uses a linear vector machine to model the relationship between input features and output traffic flow, thereby making predictions.
● ARIMA [3]: The Auto-Regressive Integrated Moving Average model, combined with the Kalman filter, is employed for time series forecasting.
● FNN [23]: A Feedforward Neural Network with two hidden layers and L2 regularization, used for learning complex patterns in the traffic data.
● FC-LSTM [23]: A Recurrent Neural Network architecture that integrates Fully Connected Long Short-Term Memory (LSTM) units for capturing temporal dependencies in traffic flow data.
● DCRNN [22]: The Diffusion Convolutional Recurrent Neural Network (DCRNN) leverages diffusion convolutional networks to learn spatial information, coupled with a sequence-to-sequence model to capture temporal dynamics.
● STGCN [36]: The Spatio-Temporal Graph Convolutional Network (STGCN) combines graph convolution with 1D convolution to model both spatial and temporal dependencies in traffic flow.
● ASTGCN [33]: The Attention-based Spatio-Temporal Graph Convolutional Network (ASTGCN), which employs an attention mechanism to enhance the learning of spatio-temporal relationships in traffic data.
● STSGCN [20]: The Spatial-Temporal Synchronous Graph Convolutional Network (STSGCN), which captures both spatial and temporal characteristics by stacking multiple local Graph Convolutional Network (GCN) layers along the temporal dimension.
● T-GCN [44]: The T-GCN framework integrates Graph Convolutional Networks (GCNs) with Gated Recurrent Units (GRUs), where GCNs learn the spatial topology of the road network and GRUs capture the temporal dependencies in traffic data.
● Graph WaveNet [32]: Graph WaveNet constructs adaptive adjacency matrices that preserve implicit spatial relationships while also designing a framework for efficiently capturing spatio-temporal dependencies through the fusion of dilated causal convolutions with graph convolutions.
● MRA-BGCN [45]: The Multi-Scale Residual Attention-based Bi-directional Graph Convolution Network (MRA-BGCN) builds node graphs based on road network distances and edge graphs based on edge interaction patterns. It models node and edge correlations separately using bicomponent graph convolution.
● TASSGN [39]: A time-aware structural semantic coupled graph network (TASSGN) is proposed to learn both structural and semantic features of graphs by designing a new graph learning module, proposing a self-sampling method and a time-aware graph encoder to capture temporal features, and generating sparse graphs to capture node unique features.
● MegaCRN [40]: A recurrent network that utilizes a memory network. Incorporate the concept into the Meta-Graph Convolutional Recurrent Network (MegaCRN) by integrating a Meta-Graph Learner, which is enhanced by a Meta-Node Bank, into the GCRN encoder-decoder framework.
● LEISN-ED [41]: This paper proposes a Long-term Explicit–Implicit Spatio-Temporal Network (LEISN) for traffic flow prediction, which includes a Long-term Dependency Module to store hidden states from multiple previous time steps and two graph convolution-based branches to extract explicit and implicit spatial features, with all features fused to predict the next state.

### 4.4 Experimental results

The performance of the STIL-TA model was compared against 16 commonly used baseline models in forecasting tasks over 15, 30, and 60-minute time horizons. Compared to the best baseline model, STIL-TA also demonstrated improvements in predictions for other time steps. The experimental results shown in Table 2 and Table 3 indicate that the proposed STIL-TA model nearly achieved optimal predictive performance on the four datasets. For instance, in the 15-minute and 60-minute prediction tasks, STIL-TA outperformed the state-of-the-art MegaCRN model by 5.42% and 6.77% in terms of MAE and 0.37% and 4.19% in terms of RMSE, respectively.

**Table 2:** Performance comparison of different traffic flow prediction models on METR-LA and PEMS-BAY datasets.

Data	Models	15min			30min			60min		
		MAE	RMSE	MAPE	MAE	RMSE	MAPE	MAE	RMSE	MAPE
METR-LA	HA	4.16	7.80	13.00%	4.16	7.80	13.00%	4.16	7.80	13.00%
	VAR	4.42	7.89	10.20%	5.41	9.13	12.7%	6.52	10.11	15.80%
	SVR	3.99	8.45	9.30%	5.05	10.87	12.10%	6.72	13.76	16.70%
	ARIMA	3.99	8.21	9.60%	5.15	10.45	12.70%	6.90	13.23	17.40%
	FNN	3.99	7.94	9.90%	4.23	8.17	12.90%	4.49	8.69	14.00%
	FC-LSTM	3.44	6.30	9.60%	3.77	7.23	10.90%	4.37	8.69	13.20%
	DCRNN	2.77	5.38	7.30%	3.15	6.45	8.80%	3.60	7.59	10.50%
	STGCN	2.88	5.74	7.62%	3.47	7.24	9.57%	4.59	9.40	12.70%
	ASTGCN	4.86	9.27	9.21%	5.43	10.61	10.13%	6.51	12.52	11.64%
	STSGCN	3.31	7.62	8.06%	4.13	9.77	10.29%	5.06	11.66	12.91%
	T-GCN	3.03	5.26	7.81%	3.52	6.12	9.45%	4.30	7.31	11.8%
	Graph WaveNet	2.69	5.15	6.90%	3.07	6.22	8.37%	3.53	7.37	10.01%
	MRA-BGCN	2.67	5.12	6.80%	3.06	6.17	8.30%	3.49	7.30	10.00%
	TASSGN	2.72	5.27	7.14%	3.03	**6.05**	8.22%	**3.39**	**7.01**	**9.78%**
	MegaCRN	**2.55**	5.18	**6.40%**	3.06	6.12	**8.03%**	3.44	7.35	9.85%
	LEISN-ED	2.77	5.29	7.18%	3.13	6.33	8.48%	3.52	7.40	9.97
	**STIL-TA**	2.65	**5.10**	6.95%	**3.02**	6.26	8.41%	3.44	7.29	10.11%
PEMS-BAY	HA	2.88	5.59	6.80%	2.88	5.59	6.80%	2.88	5.59	6.80%
	VAR	1.74	3.16	3.60%	2.32	4.25	5.00%	2.93	5.44	6.50%
	SVR	1.85	3.59	3.80%	2.48	5.18	5.50%	3.28	7.08	8.00%
	ARIMA	1.62	3.30	3.50%	2.33	4.76	5.40%	3.38	6.50	8.30%
	FNN	2.20	4.42	5.19%	2.30	4.63	5.43%	2.46	4.98	5.89%
	FC-LSTM	2.05	4.19	4.80%	2.20	4.55	5.20%	2.37	4.96	5.70%
	DCRNN	1.38	2.95	2.90%	1.74	3.97	3.90%	2.07	4.47	4.90%
	STGCN	1.36	2.96	2.90%	1.81	4.27	4.17%	2.49	5.69	5.79%
	ASTGCN	1.52	3.13	3.22%	2.01	4.27	4.48%	2.61	5.42	6.00%
	STSGCN	1.44	3.01	3.04%	1.83	4.18	4.17%	2.26	5.21	5.40%
	T-GCN	1.50	2.83	3.14%	1.73	**3.40**	3.76%	2.18	4.35	4.94%
	Graph WaveNet	1.30	2.74	2.73%	1.63	3.70	3.67%	1.95	4.52	4.63%
	MRA-BGCN	1.29	2.72	2.90%	1.61	3.67	3.80%	1.91	4.46	4.60%
	TASSGN	1.29	2.73	2.71%	1.59	3.60	3.58%	1.86	4.34	4.32%
	MegaCRN	1.29	2.72	2.70%	1.62	3.74	3.69%	1.92	4.53	4.62%
	LEISN-ED	1.36	2.81	2.86%	1.68	3.74	3.81%	1.98	4.52	4.67%
	**STIL-TA**	**1.22**	**2.71**	**2.63%**	**1.55**	3.58	**3.53%**	**1.79**	**4.24**	**4.27%**

**Table 3:** Performance comparison of different traffic flow prediction models on PEMS04 and PEMS08 datasets.

Data	Models	15min			30min			60min		
		MAE	RMSE	MAPE	MAE	RMSE	MAPE	MAE	RMSE	MAPE
PEMS04	HA	28.92	42.69	20.31%	33.73	49.37	24.01%	46.97	67.43	35.11%
	VAR	21.94	34.30	16.42%	23.72	36.58	18.02%	26.76	40.28	20.94%
	SVR	22.52	35.30	14.71%	27.63	42.23	18.29%	37.86	56.01	26.72%
	ARIMA	31.05	45.22	12.36%	33.37	48.80	24.18%	36.58	51.56	26.29%
	FC-LSTM	21.42	33.37	15.32%	25.83	39.10	20.35%	36.41	50.73	29.92%
	DCRNN	20.34	31.94	13.65%	23.21	36.15	15.70%	29.24	44.81	20.09%
	STGCN	19.35	30.76	12.81%	21.85	34.43	14.13%	26.97	41.11	16.84%
	ASTGCN	20.15	31.43	14.03%	22.09	34.34	15.47%	26.03	40.02	19.17%
	STSGCN	19.41	30.69	12.82%	21.83	34.33	14.54%	26.27	40.11	14.71%
	Graph WaveNet	18.15	29.24	12.27%	19.12	30.62	13.28%	20.69	33.02	14.11%
	**STIL-TA**	**17.96**	**28.55**	**12.04%**	**18.88**	**29.89**	**13.11%**	**20.13**	**32.47**	**13.96%**
PEMS08	HA	23.52	34.96	14.72%	27.67	40.49	17.37%	39.28	56.74	25.17%
	VAR	19.52	29.73	12.54%	22.25	33.30	14.23%	26.17	38.97	17.32%
	SVR	17.93	27.69	10.95%	22.41	34.53	13.97%	32.11	47.03	20.99%
	ARIMA	30.15	42.56	20.98%	31.09	44.32	22.73%	34.52	46.78	24.69%
	FC-LSTM	17.38	26.27	12.63%	21.22	31.97	17.32%	30.69	43.96	25.72%
	DCRNN	15.64	25.48	10.04%	17.88	27.63	11.38%	22.51	34.21	14.17%
	STGCN	15.30	25.03	9.88%	17.69	27.27	11.03%	25.46	33.71	13.34%
	ASTGCN	16.48	25.09	11.03%	18.66	28.17	12.23%	22.83	33.68	15.24%
	STSGCN	15.45	24.39	10.22%	16.93	26.53	10.84%	19.50	30.43	12.27%
	Graph WaveNet	14.02	22.76	8.95%	15.24	24.22	9.57%	16.67	26.77	10.86%
	**STIL-TA**	**13.86**	**22.04**	**8.88%**	**14.96**	**23.79**	**9.23%**	**16.12**	**25.36**	**10.21%**

It is evident that statistical methods (HA, VAR, ARIMA) and traditional machine learning models such as SVR and FC-LSTM did not perform as well, consistently falling short of the deep learning-based approaches. SVR and FC-LSTM only consider temporal features, failing to effectively capture spatial dependencies, which are crucial for spatiotemporal traffic forecasting. Consequently, their predictive performance was suboptimal. Although the VAR model can represent spatial and temporal correlations across different time series, its ability to model nonlinear and dynamic spatiotemporal dependencies is limited, leading to poor performance in spatiotemporal traffic forecasting tasks.

GCN based models, on the other hand, are capable of handling non-Euclidean traffic data and effectively capturing hidden relationships between road network nodes. Therefore, spatiotemporal GCN models such as STGCN and STSGCN performed well in the experiments. Despite STSGCN’s ability to simultaneously capture spatiotemporal features, it uses a simple sliding window to model temporal dependencies, neglecting the fine-grained temporal patterns, which resulted in suboptimal performance. Attention-based models, such as ASTGCN, performed relatively well due to their flexibility in capturing temporal dependencies within the sequence.

RNN based methods are limited in their ability to capture long-term temporal dependencies. In contrast, the STIL-TA model significantly outperformed RNN based models, especially in long-term prediction tasks. STGCN and Graph WaveNet are two classic CNN based spatiotemporal models. They model temporal correlations using 1D CNNs or TCNs along the time dimension, employing small convolutional kernels to capture local features. However, these models face challenges in long-term predictions due to their inability to effectively capture long-range temporal information. Compared to Graph WaveNet, our method demonstrated more accurate long-term predictions, while the short-term performance was similar. This can be attributed to the fact that spatial and temporal dependencies are often more stable in short-term predictions, and Graph WaveNet lacks an attention mechanism to further exploit spatiotemporal features. In contrast, STIL-TA leverages attention mechanisms to focus on relevant information from each time slice in a data-driven manner, effectively capturing long-term temporal dependencies.

In general, as the prediction horizon increases, model performance tends to be influenced by more complex and uncertain factors. However, the STIL-TA model exhibited only a marginal decline in performance for long-term predictions, indicating its robustness in handling complex situations, particularly in long-term forecasting tasks. The experimental results validate the effectiveness of STIL-TA in capturing dynamic spatial dependencies and long-term temporal dependencies. Furthermore, they demonstrate that STIL-TA can uncover hidden dynamic associations between road network nodes, thus effectively capturing spatial correlations. Although the difficulty of prediction tasks increases with the forecast horizon, as shown in Table 2, STIL-TA still outperformed other models in long-term predictions, further validating the effectiveness of STIL-TA’s interactive learning strategy.

Based on the experimental results in the Table 3, we can analyze the performance of different traffic flow prediction models on the PEMS04 and PEMS08 datasets. First, the STIL-TA model performs well on all time intervals (15, 30 and 60 minutes) and both datasets, and its MAE, RMSE and MAPE metrics are significantly lower than those of the other models, which shows its superiority in capturing the spatio-temporal characteristics of traffic flow. On the PEMS08 dataset, the STIL-TA model also performs the best in all prediction intervals. For example, at the 15-minute prediction interval, it has the lowest MAE of 13.86, RMSE of 22.04, and MAPE of 8.88%. These results indicate that the STIL-TA model not only performs well in short-term forecasting, but also has high accuracy and stability in long-term forecasting. In contrast, other models such as HA, VAR, SVR, and other traditional methods do not perform as well as STIL-TA for all prediction intervals and datasets. Even some deep learning-based models, such as Graph WaveNet and ASTGCN, although they perform better in some cases, they are still inferior to the STIL-TA model in general.

In summary, the STIL-TA model effectively improves the accuracy of traffic flow prediction through its interactive learning and temporal attention mechanisms, and especially performs well in dealing with complex spatio-temporal dependencies. These results indicate that the STIL-TA model has great potential for application in practical traffic management and can provide more reliable support for traffic flow prediction.

**Fig 3:**
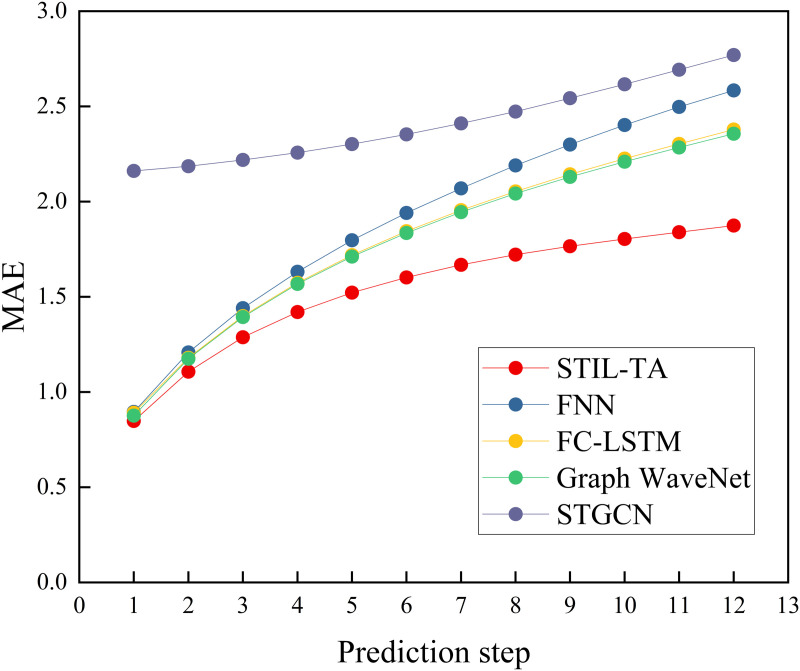
Visual comparison of different error metrics (MAE).

**Fig 4:**
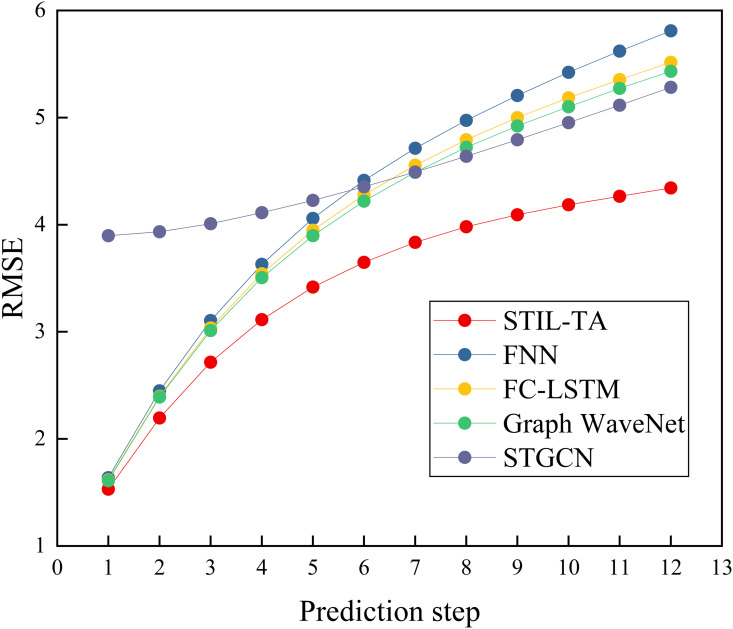
Visual comparison of different error metrics (RMSE).

**Fig 5:**
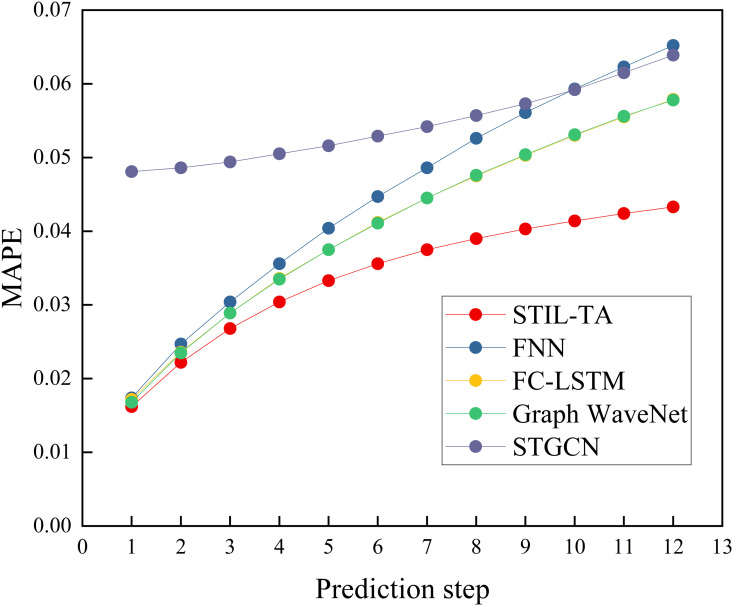
Visual comparison of different error metrics (MAPE).

To provide a clearer illustration of the advantages of the STIL-TA model, we have visualized the experimental results of STIL-TA compared to FNN, FC-LSTM, Graph WaveNet, and STGCN on the PEMS-BAY dataset, as shown in Fig 3–4 and Fig 5. The results clearly demonstrate that STIL-TA significantly outperforms FNN, FC-LSTM, Graph WaveNet, and STGCN in terms of prediction performance. This indicates that the proposed model is more effective at capturing the dynamic spatiotemporal characteristics of traffic flow. Further analysis reveals that as the prediction horizon increases, the growth of prediction errors remains relatively small. When the prediction horizon exceeds 15 minutes, the prediction error of STIL-TA consistently remains significantly lower than that of the other models, further validating the superior performance of this model in long-term predictions.

### 4.5 Ablation study

**Fig 6:**
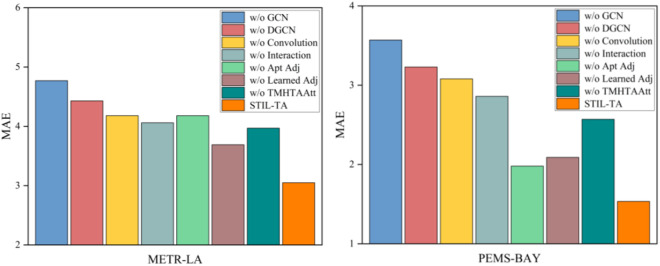
Visualization of MAE metrics on METR-LA and PEMS-BAY datasets.

**Fig 7:**
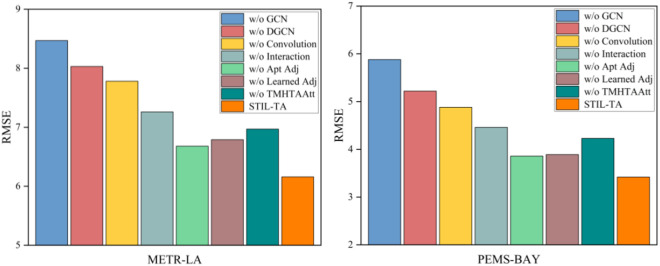
Visualization of RMSE metrics on METR-LA and PEMS-BAY datasets.

**Fig 8:**
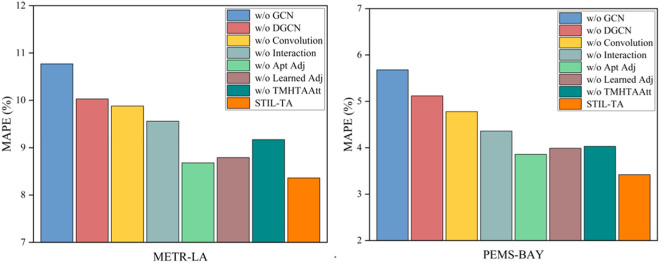
Visualization of MAPE metrics on METR-LA and PEMS-BAY datasets.

To further investigate the performance of various modules within the STIL-TA model, this study designed seven variants of the STIL-TA model. The performance of these variants was evaluated based on experiments conducted on the METR-LA and PEMS-BAY datasets. Specifically, we computed and visualized the performance of these eight variants in terms of MAE, RMSE, and MAPE, as shown in Fig 6–7 and Fig 8. The differences between each variant and the original STIL-TA model are as follows:

● w/o GCN: This variant is based on STIL-TA but removes the diffusion GCNs module.
● w/o DGCN: This variant replaces the DGCN module in STIL-TA with a standard diffusion GCN, using a predefined initial adjacency matrix as the input to the GCN.
● w/o Conv: This variant is based on STIL-TA but eliminates the 1D convolution module from the interactive learning structure.
● w/o Interaction: This variant replaces the interactive learning structure in STIL-TA with a temporal convolutional network (TCN) module, which is concatenated with a dynamic convolution module. The TCN consists of 6 layers, with 64 feature channels.
● w/o Apt Adj: This variant is based on STIL-TA but removes the adaptive adjacency matrix from the DGCN module, replacing it with a predefined initial adjacency matrix as the input to the graph generator.
● w/o Learned Adj: This variant is based on STIL-TA but removes the graph generator structure, retaining the adaptive adjacency matrix while replacing the fusion GCN in the DGCN module with a diffusion GCN.
● w/o TMHTAAtt: This variant is based on STIL-TA but removes the temporal multi-head trend-aware self-attention (TMHTAAtt) module.

The graph convolutional network plays a crucial role in the STIL-TA model. Furthermore, the proposed IDGCN and TMHTAAtt modules are also critical to enhancing the overall model performance. Specifically, the 1D convolution is essential for expanding the receptive field and serves as a core component in the interactive learning structure. The results of the ablation study indicate that 1D convolution significantly improves the model’s performance.

Moreover, an ablation analysis was performed on the two adjacency matrices defined within the DGCN module in the STIL-TA model. As shown in Fig 6, Fig 7 and Fig 8, the adaptive adjacency matrix is crucial for the model’s prediction accuracy. The combination of the learnable adjacency matrix and the adaptive adjacency matrix generates a dynamic adjacency matrix. Further analysis revealed that this dynamic adjacency matrix enables the graph convolution to better capture the hidden spatial correlations within traffic data, thereby validating the effectiveness of the two core structures: interactive learning and dynamic graph convolution. Finally, the TMHTAAtt mechanism effectively captures local context, fully exploits the spatiotemporal information of traffic flow, and captures dynamic temporal relationships, which is essential for the STIL-TA model.

### 4.6 Visualization analysis

**Fig 9:**
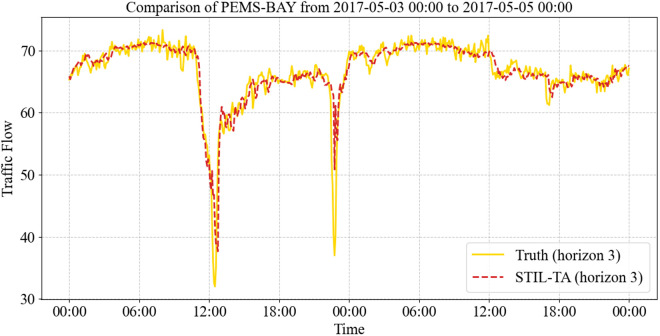
Visualization of the true and predicted values of the STIL-TA model on the PEMS-BAY dataset (Horizon 3).

**Fig 10:**
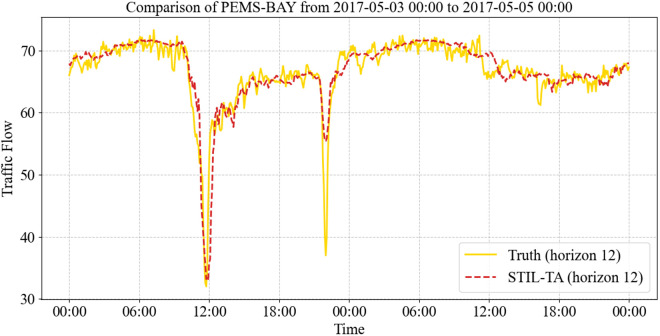
Visualization of the true and predicted values of the STIL-TA model on the PEMS-BAY dataset (Horizon 12).

Furthermore, Fig 9 and Fig 10 presents a visualization of the true and predicted traffic flow values at specific time intervals for STIL-TA on PEMS-BAY, focusing on the 15-minute (Horizon 3) and 60-minute (Horizon 12) prediction horizons. It is evident that the STIL-TA model accurately captures peak periods and overall traffic patterns, effectively predicting the fluctuations in traffic flow.

Additionally, the results demonstrate that the 15-minute predictions are more accurate than the 60-minute predictions. This discrepancy can be attributed to the complex dynamic spatiotemporal characteristics of traffic flow, which make long-term predictions inherently more challenging. Nevertheless, STIL-TA’s 60-minute predictions still closely follow the true traffic fluctuations, further validating the accuracy and effectiveness of the proposed model in traffic flow forecasting.

### 4.7 Comparison of time complexity

As shown in Table 4, we compare the training and inference times of various models on the METR-LA dataset. During the training phase, STGCN demonstrates the fastest speed; however, its performance in practical predictions is suboptimal. In contrast, DCRNN exhibits a significantly slower training speed due to its reliance on the RNN structure, which requires additional time to learn temporal features of the time series data. In the inference phase, STIL-TA achieves the fastest inference time. On the other hand, both DCRNN and STGCN have relatively slower inference speeds, primarily because they necessitate multiple iterative computations to generate predictions. STIL-TA and Graph WaveNet benefit from shorter inference times, as they are capable of generating 12-step predictions in a single computation. While STIL-TA incurs slightly higher computational costs during the training phase compared to STGCN, it consistently outperforms other models in terms of traffic flow prediction accuracy.

**Table 4:** Computation time comparison with other models on the PEMS-BAY dataset.

Data	Models	Computation Time	
		Training (s)/epoch	Inference(s)
METR-LA	DCRNN	650.64	110.52
	STGCN	51.35	94.56
	Graph WaveNet	182.21	6.55
	STIL-TA	136.55	5.91

In terms of trade-off considerations for model deployment, STIL-TA, although the training phase is time-consuming (136.55s/epoch) compared to STGCN (51.35s/epoch), this computational cost enhancement leads to significant accuracy gains (15.49% reduction in MAE). Notably, STIL-TA exhibits the best real-time performance in the inference phase (a single computation generates a 12-step prediction), which makes it particularly suitable for real-world traffic system scenarios with stringent timeliness requirements, e.g., adaptive signal control (200ms-level response). The model design achieves a balance between accuracy and efficiency through three strategies: (1) the training phase uses a parallel computing architecture for dynamic graph learning, which increases the initial training burden but avoids the sequential computation bottleneck of the RNN structure; (2) the inference phase utilizes the single feed-forward property of the spatio-temporal attention mechanism to reduce the computational complexity while maintaining the prediction accuracy; and (3) the modular design allows for the deployment of the model on the resource-constrained edge devices to deploy only critical computational paths (e.g., shedding interaction learning branches).

## 5 Conclusion

This paper presents STIL-TA, an efficient and accurate traffic flow prediction model that integrates non-Euclidean traffic flow characteristics with an interactive learning strategy and temporal multi-head trend-aware self-attention (TMHTAAtt). By capturing dynamic spatiotemporal features, STIL-TA addresses key challenges of traditional models, such as limited interaction, incomplete spatiotemporal feature capture, and difficulties in long-term forecasting. The model generates dynamic graph structures from spatiotemporal data and uses a predefined adjacency matrix to model dynamic node dependencies, uncovering hidden spatial correlations. The incorporation of dynamic graph convolutional networks within an interactive framework allows STIL-TA to capture periodic trends and spatiotemporal dependencies. Additionally, the TMHTAAtt mechanism enhances its ability to identify dynamic temporal patterns, improving prediction accuracy. Experimental results demonstrate that STIL-TA outperforms existing methods on real-world traffic datasets, validating its effectiveness.

This study has achieved significant results in modeling the spatio-temporal dynamics of traffic flow, but it is still complicated by external factors such as weather, special events and holidays. In this paper, we will consider the effects of external factors (e.g., weather and social events) on traffic flow prediction in the next step of the study and explore the application of STIL-TA on large-scale datasets to further enhance its prediction capability.
